# Magnesium sulfate has sex-specific, dose-dependent vasodilator effects on preterm placental vessels

**DOI:** 10.1186/s13293-015-0040-z

**Published:** 2015-11-04

**Authors:** Clint Gray, Mark H. Vickers, Rebecca M. Dyson, Clare M. Reynolds, Mary J. Berry

**Affiliations:** Department of Paediatrics and Child Health, University of Otago, Wellington, New Zealand; Centre for Translational Physiology, University of Otago, Wellington, New Zealand; Gravida: National Centre for Growth and Development, Liggins Institute, University of Auckland, Auckland, New Zealand; Department of Paediatrics, Graduate School of Medicine and IHMRI, University of Wollongong, Wollongong, NSW Australia; Capital and Coast District Health Board, Wellington, New Zealand

## Abstract

**Background:**

Women at risk of preterm delivery receive magnesium sulfate (MgSO_4_) in the pre-delivery phase to reduce their child’s risk of neurodevelopmental complications associated with preterm birth. However, the mechanisms underpinning its placental vascular role remain uncertain.

**Methods:**

The aim of this study was to examine MgSO_4_ action on vascular tone in male and female human placental vessels from term and preterm deliveries. Vessels were obtained from placental biopsy following birth at term (37–41 weeks) or preterm gestation (<36 weeks of gestation). The vessels were mounted on a pressure myograph, pre-constricted with synthetic endoperoxide prostaglandin PGH_2_ (U46619) (0.1–100 μmol/l), and percentage of relaxation was calculated following incubation with bradykinin. Experiments were carried out in the presence of MgSO_4_ (0.2 mmol/l), N_Ψ_-nitro-L-arginine methyl ester (L-NAME) (0.1 mmol/l), indomethacin (10 μmol/l), Ca^2+^-activated K^+^ channel blocker TRAM-34 (1 μM) and apamin (3 μM) to assess mechanisms of vascular function. Vascular [calcium ions (Ca^2+^)] was analysed using a colorimetric calcium assay.

**Results:**

Vasodilation in vessels from preterm males was significantly blunted in the presence of MgSO_4_ when compared to preterm female and term male and female vessels. Overall, MgSO_4_ was observed to differentially modulate placental vascular tone and vascular calcium concentrations in a sex-specific manner.

**Conclusions:**

As MgSO_4_ regulates human placental blood flow via specific pathways, foetal sex-specific MgSO_4_ treatment regimes may be necessary. In an era of increasing awareness of individualised medicine, sex-specific effects may be of importance when developing strategies to optimise care in high-risk patients.

## Background

Preterm birth is increasingly common, with recent global estimates suggesting that as many as 10 % of infants (approximately 15 million infants per annum) are born prior to 37 weeks gestation [1, 2]. Although survival rates for preterm infants have improved, rates of cerebral palsy (CP), neurodevelopmental delay and cognitive and behavioural or psychiatric issues are far more prevalent in infants born preterm when compared to term-born infants [3, 4]. Although much attention has focused on infants born at the extremes of gestation, there appears to be an effect of maturity at birth on later neurological function that extends the full spectrum of gestational age [5, 6]. 

CP is a permanent disorder of motor function due to disrupted or altered brain development. Its functional manifestations may evolve with time but there are no curative therapies currently available. The costs of CP are significant across a personal as well as global public health economic platform [7, 8]. In an effort to reduce CP risk in children born prematurely, obstetric practice now includes treatment with intravenous magnesium sulphate (MgSO_4_) for women at high risk of delivery before 30 weeks gestation [9]. While the overall efficacy of this approach has been well described, the number of women that needed treatment to prevent one case of CP is relatively high [10]. This suggests that MgSO_4_ either has an indirect role in the prevention of CP or that its effects are most beneficial to a subgroup of patients. Male disadvantage in survival and neurological outcomes in preterm infants has consistently been demonstrated [11, 12]. However, the neuroprotective effects of antenatal MgSO_4_, in terms of sex specificity, have not yet been reported.

MgSO_4_ is a modulator of vascular tone [13, 14]. Therefore, the neuroprotective effects of MgSO_4_ may be partially mediated via improved placental perfusion, with improved nutrient transfer to the foetus, especially within the context of preterm labour. Maturational and sex-specific differences in vascular flow have already been identified in preterm infants. Newborn preterm males demonstrate increased microcirculatory flow compared to either preterm females or term-born infants of either sex [15]. Therefore, perinatal regulation of vascular tone may differ according to either gestational age and/or sex.

MgSO_4_ has been shown to freely cross the blood-brain barrier and maternal-placental-foetal interface. In adults, the physiological concentration of serum magnesium is 1.5 to 2.5 mEq/l (1.8 to 3.0 mg/dl), with approximately half inactivated, through binding to plasma proteins [14, 16, 17]. Therapeutic concentrations of maternal MgSO_4_ recommended for foetal neuroprotection are 1.8–3.5 mEq/l (2.1 to 4.0 mg/dl). It has been shown that following maternal MgSO_4_ treatment, Mg serum concentrations increase within 60 min and amniotic fluid concentrations increase after approximately 3 h [18]. Furthermore, foetal and maternal serum concentrations correlate well with foetal concentrations being elevated 24 h following maternal administration [19]. Despite well-established treatment regimes, the mechanisms governing MgSO_4_ and subsequent placental vascular function have yet to be defined. In the current study, experimental concentrations of MgSO_4_ were determined following a series of pilot studies in which the in vitro vasodilatory effects of MgSO4 were tested to the point of saturation where no further vasodilation was observed. Our study used a final vessel bath concentration of 3.6 mg/dl of MgSO_4_ as this was both within the therapeutic target range and had been shown to induce changes in vascular tone in vitro.

There are very few studies characterising the direct cardiovascular effects of MgSO_4_. In instrumented animal models, pregnant ewes treated with MgSO_4_ during late gestation have a reduction in maternal mean arterial pressure, increased uterine artery blood flow and increased foetal oxygenation, without an increase in foetal heart rate or blood pressure [20, 21]. However, a paucity of data exists with regard to the effects of MgSO_4_ on placental vascular function, and no studies have investigated the effect of MgSO_4_ on preterm human placental vasculature function.

Furthermore, the placenta is often generalised as an asexual organ and any influence of placental sex is routinely overlooked. However, the human placenta has distinct sexually dimorphic function. Male and female placentae have been shown to exhibit differences in global gene, protein expression, immune function, steroid profiles, growth factors, structure and overall function  [22, 23, 24, 25, 26]. Sex-specific differences in vasomotor function may therefore be apparent in the placental vasculature as well as in the new born preterm human. The current study has used pressure myography to investigate the hypothesis that, in human placental chorionic plate vessels, there are sexually dimorphic differences in placental vascular function following MgSO_4_ treatment.

## Methods

Approval for the study was granted by the University of Otago Human Ethics Committee (H13/058).

### Patient eligibility

Eligible women admitted to the Delivery Suite of Wellington Hospital, Capital and Coast District Health Board, New Zealand, during the study period were approached for enrolment in the study. Eligible women were (i) carrying a singleton foetus with no known congenital infection or malformation or genetic or chromosomal abnormalities, (ii) of known gestational age (confirmed first trimester dating scan), (iii) no maternal history of pre-eclampsia or foetal growth restriction and (iv) able to read a patient information sheet and give written informed consent. The term cohort was comprised of women delivering between 37 and 41 weeks of gestation. The preterm cohort was comprised of women delivering at less than 36 weeks of gestation who had not received MgSO_4_ treatment prior to birth. Gestational age was ascertained by the mothers’ attending lead maternity carer or admitting consultant obstetrician. All patients received obstetric care in accordance with routine clinical practice, and members of the study team were not involved in any aspect of the clinical care of any of the study subjects. The diagnostic criteria for the patients study eligibility was confirmed by an independent obstetrician following assessment at the time of admission and delivery. Term and preterm cohorts were further divided by infant sex (i.e. male term, female term, male preterm and female preterm) before statistical analysis. All maternal data were collected from patient records at the point of delivery.

### Sample collection

Following delivery, the placentae were placed on ice pending biopsy collection. All biopsies were kept on ice for no longer than 15 min following delivery to dissection and myography cannulation. A 1-cm^3^ placental biopsy was obtained using a placental measuring gauge to ensure all biopsies were collected from the same approximate region of the chorionic foetal side of the placenta. The biopsies were then placed in an ice-cold tissue collection buffer (TCB) (119 mM NaCl, 4.7 mM KCl, 1.6nM CaCl_2_, 2H_2_0, 24 NaHCO_3_, 1.18 KH_2_PO_4_, 1.2 MgSO_4_, 0.05 EDTA, 5.5 glucose at pH 7.4) and remained on ice until studied.

### Vascular studies

All chemicals and vasoactive drugs were purchased from Sigma-Aldrich (Auckland, NZ) unless stated otherwise. In brief, intact placental chorionic plate vessels (diameter 212 ± 19 μm) were isolated from the placental biopsy and any remaining connective tissue removed under a dissecting microscope. The vessel segments were then mounted on a pressure myograph system (Living Systems Instrumentation, Burlington, VT, USA). The foetal vessels were attached to two glass microcannulae and secured with nylon thread sutures and then further aligned, so as not to stretch the vessel.

The vessel segments were pressurised to 70 mmHg following the equilibration period of 30 min or cessation of vascular activity at 37 °C in TCB gassed with a mixture of 95 % O_2_ and 5 % CO_2_. The vessels were allowed to equilibrate under these conditions for 45 min. Thereafter, cumulative concentration response curves were constructed for the synthetic analogue endoperoxide prostaglandin H2 (PGH_2_), U46619 (0.1–100 nmol/l). Changes in diameter at each U46619 concentration were compared to the initial vessel diameter as percent constriction and then normalised as percent maximum constriction. Following pre-constriction with U46619 −log concentration equal to the equivalent pEC80 (concentration equal to 80 % of maximal response) and cumulative concentration curves were constructed using the endothelium-dependent vasodilator bradykinin (10 ρmol/l–1 μmol/l). Following the initial experiments, bradykinin was used in preference to acetylcholine as it consistently produced larger and reproducible responses. Changes in the vessel diameter at each concentration were compared to the vessel diameter following pre-constriction with U46619 and then normalised as percentage relaxation. Following a washout, the concentrations response curves were performed in the presence of various vascular inhibitory drugs. Preliminary experiments determined the dose-dependent effect of MgSO_4_ (0.05–0.25 mmol/l) on isolated vessels. Concentrations of MgSO_4_ were initially determined by previously reported cord blood concentrations following maternal MgSO_4_ treatment [27]. A minimum of two vessels per placenta were analysed and averaged following vascular experimental protocols. All vasoactive drugs were applied extraluminally.

To analyse mediators of endothelium-dependent relaxation in human placental chorionic plate vessels, the three main vasodilatory pathways were examined (endothelial-derived hyperpolarising factors (EDHF), nitric oxide (NO), prostacyclin (PGI_2_)). To block NO production, a non-specific NO synthase inhibitor L-NG-nitroarginine methyl ester (L-NAME; 0.1 mmol/l) was used. Indomethacin (INDO; 10 μmol/l) was used to investigate any vasodilation derived from the cyclooxygenase pathway. The role of EDHF activity in human chorionic plate vessels is to use the adenosine triphosphate (ATP)-type calcium ions (Ca2^+^)-activated K+ channel blocker apamin (3 μM) and intermediate-conductance Ca2^+^-activated K^+^ channel blocker TRAM-34 (1 μM). Inhibitors were used individually and in combination where stated.

### Vascular viability

Alignment and vessel suture integrity was tested by increasing the intraluminal pressure to 70 mmHg, with further adjustment of the cannulae until the artery was observed to be aligned. Vessel functional integrity was assessed by washing the vessel for three 90-s washes with physiological salt solution (PSS) and pre-constriction with U46619 (100 nmol/l) (pEC80). Vessels failing to reproduce consistent constrictions were considered non-viable and replaced with freshly excised vessels.

### Vascular [Ca^2+^]

The placental chorionic plate vessels were solubilised and lysate cleared from solubilised tissue samples by centrifugation (20,000×g for 10 min). [Ca^2+^] from the vessels was determined using a colorimetric Calcium Detection Kit (Abcam; Cambridge, UK) following the manufacturer’s instructions.

### Statistical analysis

All data are shown as means ± SEM unless stated otherwise. All observations were made in a minimum of two vessels per placenta, and the average responses calculated. *n* refers to the number of placenta used (two vessels per placenta). Concentration-relaxation curves were constructed using Prism software (GraphPad Software Inc., La Jolla, CA, USA.). Pressure myography and vascular calcium concentration data were analysed by using multifactorial analyses of variance (three-way ANOVA), with treatment, term and sex as the main factors. Where significant effects of factor and/or interactions were reported, Bonferroni multiple-comparison post hoc analysis was performed. *P* values, *F* values, and degrees of freedom are quoted for three-way ANOVA where appropriate. A Student *t* test was employed for the analysis of basic maternal and neonatal biometrics (Table 1). A probability of *P* < 0.05 was accepted as statistically significant. Statistical analysis was performed using SPSS version 21 (SPSS, Chicago, IL).

**Table 1:** Birth and placental weights refer to measurements obtained immediately after delivery

Characteristic	Term (39–41 weeks)	Preterm (32–<36 weeks)
	(*n* = 20)	(*n* = 14)
Maternal age (years)	31.1 ± 0.9	29.1 ± 1.5
Maternal weight (kg)	71 ± 2.5	67 ± 2.4
Maternal race or ethnic group (%)		
NZ European	73	61
Chinese	12	17
Pacific Islands	15	22
Nulliparous (%)	0	0
Parity	1.7 ± 0.15	1.8 ± 0.1
Twin gestation (%)	0	0
Smoking during pregnancy (%)	0	5 (1 patient)
Alcohol use during pregnancy (%)	0	0
MgS04 treatment during pregnancy (%)	0	0
In vitro fertilisation (%)	8	0
Infant sex		
Male	10	7
Female	10	7
Gestational age (weeks)		
Male	39^2^ ± 0^2^	33^6^ ± 1^3^
Female	39^6^ ± 0^2^	33^5^ ± 1^1^
Birth weight (g)		
Male	3579 ± 83*	2299 ± 191
Female	3434 ± 79*	1969 ± 283
Placental weight (g)		
Male	620 ± 82*	540 ± 72
Female	580 ± 79*	517 ± 91

Race or ethnic group was self-reported. *n* = 20 subjects for term pregnancies (*n* = 10 male, *n* = 10 female) and *n* = 14 subjects for preterm pregnancies (*n* = 7 male, *n* = 7 female). Data are shown as ± SEM and percentages where stated

*NS* not significant

*Denotes statistical significance (*P* < 0.05) between groups by Student *t* test

## Results

### Maternal and neonatal biometrics

There was no significant difference observed in maternal age, weight, parity, smoking and alcohol or substance use between groups (Table 1). Gestational age was significantly different between term and preterm births (*P* < 0.001). Mean gestational age in preterm males was not significantly different from preterm females (male, 33^6^ ± 1^3^ weeks vs. female, 33^5^ ± 1^1^ weeks), and no differences were observed between male and female term gestational age (male, 39^2^ ± 0^2^ weeks vs. female, 39^6^ ± 0^2^ weeks).

As expected, term male infants had a greater birth weight than female infants. Term infants had a greater birth weight than same-sex preterm infants (term male, 3579 ± 84 g vs. preterm male, 2299 ± 191 g; term female, 3434 ± 79 g vs. preterm female, 1969 ± 283 g, *P* < 0.01 for effect of sex and term vs. preterm). Term placentas were heavier than same-sex preterm placentas (term male, 620 ± 82 g vs. preterm male, 540 ± 72 g, *P* < 0.05) and term female, 580 ± 79 g vs. preterm female, 517 ± 91 g, *P* < 0.05). There was no effect of sex on placental weight within either gestational age group (Table 1). All women enrolled in the study delivered live-born singleton infants.

### Placental chorionic plate vessel responsiveness to Bradykinin

**Fig. 1:**
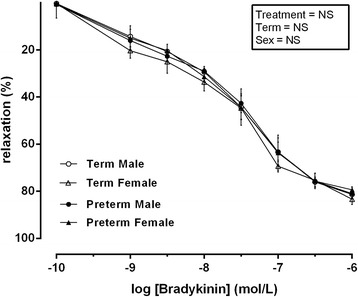
Relaxation induced by bradykinin following pre-constriction with U46619 in vessels isolated from male term pregnancies (*empty circle*) *n* = 10, female term pregnancies (*empty triangle*) *n* = 10, male preterm pregnancies (*filled circle*) *n* = 7 and female preterm pregnancies (*filled triangle*) *n* = 7. Data was analysed using a repeated measures three-way ANOVA using treatment × term × sex as the main factors. All observations were made in a minimum of two vessels per placenta, and the average responses were calculated before further statistical analysis. *n* refers to the number of placenta used. All data are presented as means ± SEM

Bradykinin (10 ρmol/l–1 μmol/l) produced a concentration-dependent vasodilation in all vessels which was not different between groups or sex within group. U46619 (0.1–100 μmol/l) produced a concentration-dependent vasoconstriction in all vessels which was not different between groups (Fig. 1).

### MgSO_4_ dose-response in placental chorionic plate vessels

**Fig. 2:**
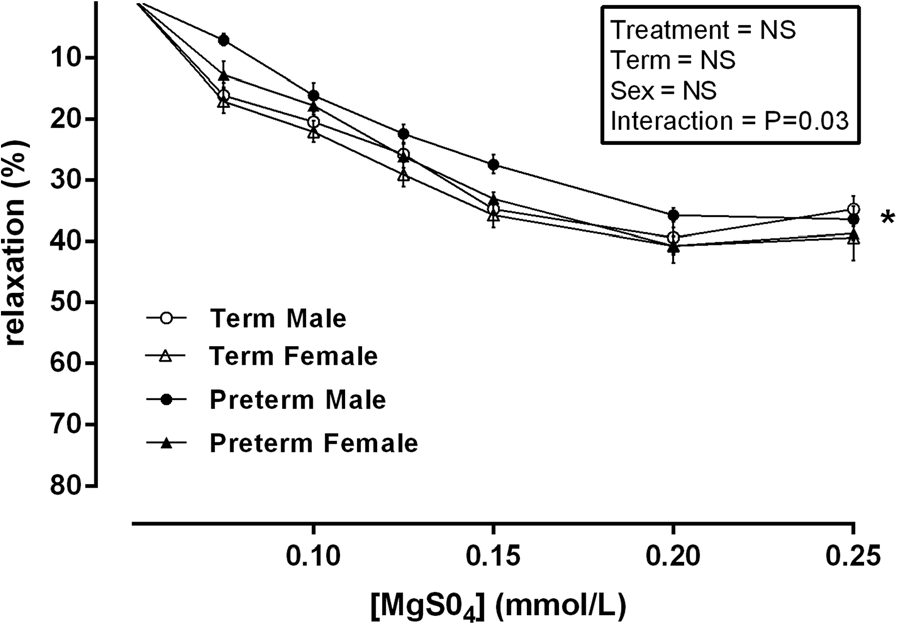
Relaxation induced by incremental concentrations of MgSO_4_ (0.05–0.25 mmol/l) in vessels isolated from male term pregnancies (*empty circle*) *n* = 10, female term pregnancies (*empty triangle*) *n* = 10, male preterm pregnancies (*filled circle*) *n* = 7 and female preterm pregnancies (*filled triangle*) *n* = 7. Data was analysed using a repeated measures three-way ANOVA using treatment × term × sex as the main factors. All observations were made in a minimum of two vessels per placenta, and the average responses were calculated before further statistical analysis. *n* refers to the number of placenta used. *Denotes *P* < 0.01 in all cases significant difference between male preterm and all other groups following post hoc analysis. All data are presented as means ± SEM

Following pre-constriction with U46619 (100 μmol/l), the vessels were treated with incremental concentrations of MgSO_4_ (0.05–0.25 mmol/l). There was no overall effect of treatment, term or sex. However, a significant interaction effect (interaction effect = treatment × sex, *P* = 0.03, *F* = 11.61, *df* = 2) was observed. This is likely due to preterm male vessels having significantly less percentage of relaxation than all other groups following MgSO_4_ administration. Following post hoc analysis, the placental chorionic plate vessels from preterm male placentae had a reduced vasodilatory response (*P* < 0.01) compared to all other groups following MgSO_4_ treatment (0.05, 0.125, 0.15 and 0.20 mmol/l). No further differences were observed at MgSO_4_ concentrations of 0.25 mmol/l as all vessels across all groups appeared to reach the point of saturation with further doses having no effect on percentage of relaxation (Fig. 2).

### Bradykinin-induced relaxation in placental chorionic plate vessels incubated with MgSO_4_

**Fig. 3:**
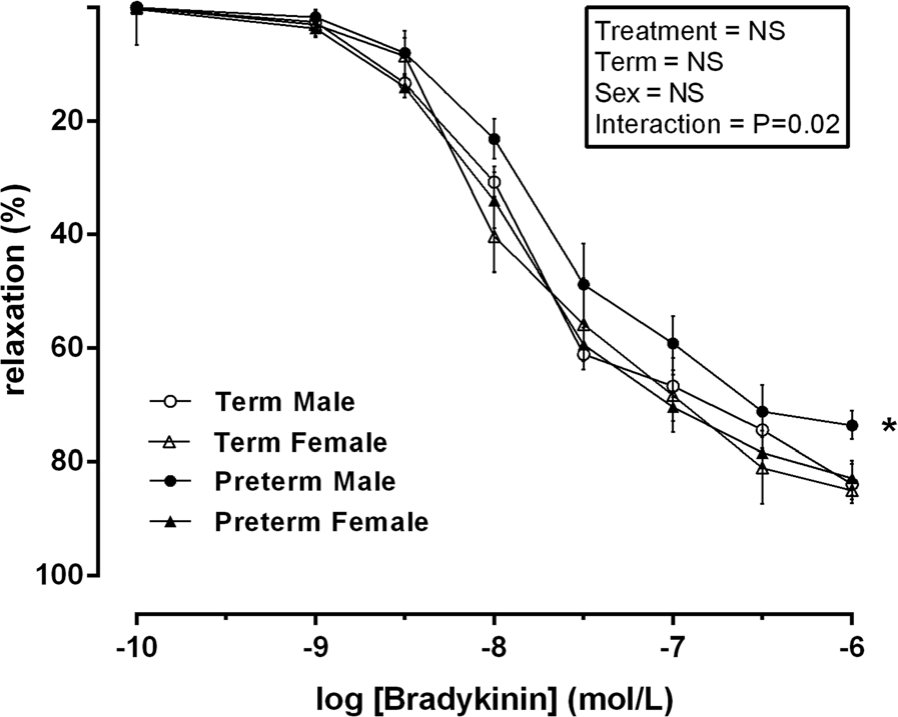
Relaxation induced by bradykinin following pre-constriction with U46619 and following incubation with MgSO_4_ (0.2 mmol/l) in vessels isolated from male term pregnancies (*empty circle*) *n* = 10, female term pregnancies (*empty triangle*) *n* = 10, male preterm pregnancies (*filled circle*) *n* = 7 and female preterm pregnancies (*filled triangle*) *n* = 7. Data was analysed using a repeated measures three-way ANOVA using treatment × term × sex as the main factors. All observations were made in a minimum of two vessels per placenta, and the average responses were calculated before further statistical analysis. *n* refers to the number of placenta used. *Denotes *P* < 0.05 in all cases significant difference between male preterm and all other groups following post hoc analysis. All data are presented as means ± SEM

Following pre-constriction with U46619 (100 μmol/l) and MgSO_4_ treatment (0.2 mmol/l), bradykinin-induced vasodilation was observed in a concentration-dependent manner in all vessels (Fig. 3). There was no overall effect of treatment, term or sex. However, a significant interaction effect (interaction effect = treatment × sex, *P* = 0.02, *F* = 9.376, *df* = 2) was observed. Following post hoc analysis, significantly less (*P* < 0.05) percentage of relaxation was observed in chorionic plate vessels from preterm male placental vessels when compared to all other groups.

### Bradykinin-induced relaxation in placental chorionic plate vessels incubated with INDO, L-NAME and MgSO_4_

**Fig. 4:**
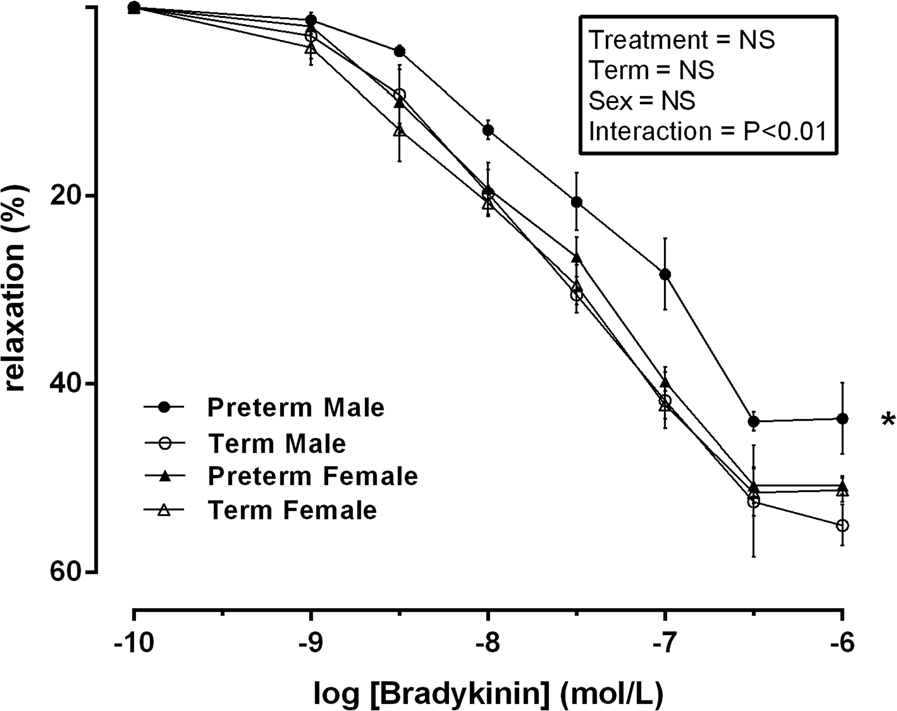
Relaxation induced by bradykinin following pre-constriction with U46619 following incubation with MgSO_4_ (0.2 mmol/l), L-NAME (0.1 mmol/l) and INDO ( μmol/l) in vessels isolated from male term pregnancies (*empty circle*) *n* = 10, female term pregnancies (*empty triangle*) *n* = 10, male preterm pregnancies (*filled circle*) *n* = 7 and female preterm pregnancies (*filled triangle*) *n* = 7. Data was analysed using a repeated measures three-way ANOVA using treatment × term × sex as the main factors. All observations were made in a minimum of two vessels per placenta, and the average responses were calculated before further statistical analysis. *n* refers to the number of placenta used. *Denotes *P* < 0.01 in all cases significant difference between male preterm and all other groups following post hoc analysis. All data are presented as means ± SEM

Following pre-constriction with U46619 (100 μmol/l) and administration of MgSO_4_ (0.2 mmol/l), INDO (10 μmol/l) and L-NAME (0.1 mmol/l), there was no overall effect of treatment, term or sex. However, a significant interaction effect (interaction effect = treatment × sex, *P* < 0.01, *F* = 8.213, *df* = 2) was observed. Following post hoc analysis, significantly less (*P* < 0.01) percentage of relaxation was significantly attenuated in male preterm placental vessels when compared to female preterm vessels and both male and female term placental vessels at all −log concentrations of bradykinin (*P* < 0.01) (Fig. 4).

### Bradykinin-induced relaxation in placental chorionic plate vessels incubated with TRAM-34, apamin, INDO and MgSO_4_

**Fig. 5:**
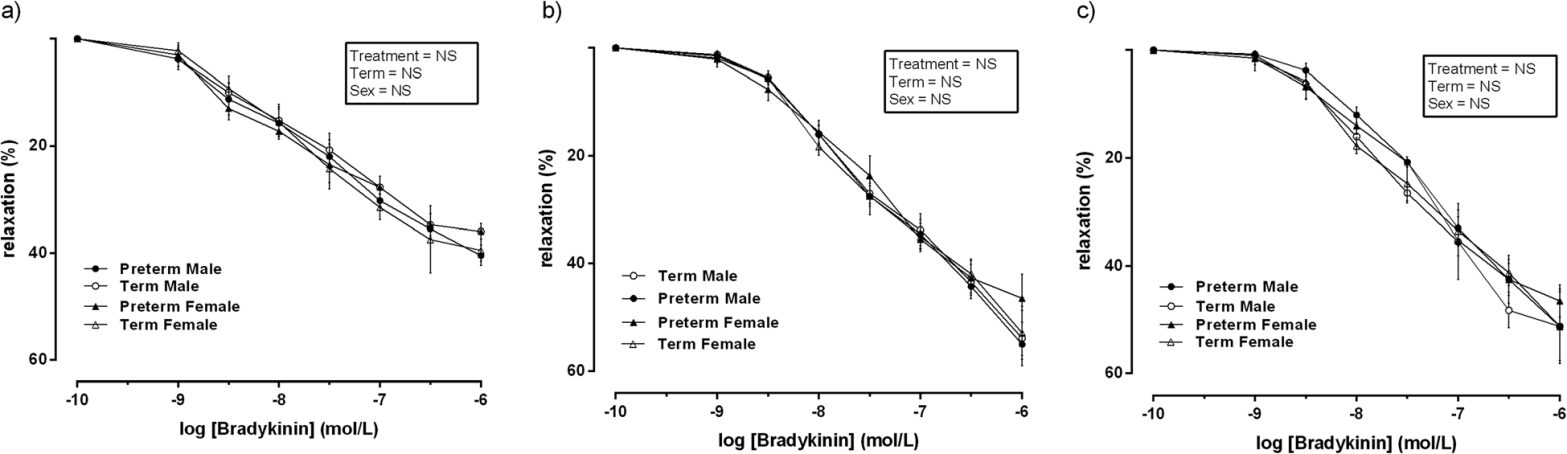
**a** Relaxation induced by bradykinin following pre-constriction with U46619 following incubation with MgSO_4_ (0.2 mmol/l), INDO (10 μmol/l), TRAM-34 (1 μM) and apamin (3 μM) in vessels isolated from male term pregnancies (*empty circle*) *n* = 10, female term pregnancies (*empty triangle*) *n* = 10, male preterm pregnancies (*filled circle*) *n* = 7 and female preterm pregnancies (*filled triangle*) *n* = 7. Data was analysed using a repeated measures three-way ANOVA using treatment × term × sex as the main factors. All observations were made in a minimum of two vessels per placenta, and the average responses were calculated before further statistical analysis. *n* refers to the number of placenta used. All data are presented as means ± SEM. **b** Relaxation induced by bradykinin following pre-constriction with U46619 following incubation with MgSO4 (0.2 mmol/l) and indomethacin (10 μmol/l) in vessels isolated from male term pregnancies (*empty circle*) *n* = 10, female term pregnancies (*empty triangle*) *n* = 10, male preterm pregnancies (*filled circle*) *n* = 7 and female preterm pregnancies (*filled triangle*) *n* = 7. Data was analysed using a repeated measures three-way ANOVA using treatment x term x sex as main factors. All observations were made in a minimum of two vessels per placenta and the average responses calculated before further statistical analysis. *n* refers to the number of placenta used. All data are means ± SEM. **c** Relaxation induced by bradykinin following pre-constriction with U46619 following incubation with MgSO_4_ (0.2 mmol/l), TRAM-34 (1 μM) and Apamin (3 μM) in vessels isolated from male term pregnancies (*empty circle*) *n* = 10, female term pregnancies (*empty triangle*) *n* = 10, male preterm pregnancies (*filled circle*) *n* = 7 and female preterm pregnancies (*filled triangle*) *n* = 7. Data was analysed using a repeated measures three-way ANOVA using treatment × term × sex as the main factors. All observations were made in a minimum of two vessels per placenta and the average responses calculated before further statistical analysis. *n* refers to the number of placenta used. All data are presented as means ± SEM

In the presence of MgSO_4_ (0.4 mmol/l), TRAM-34 (1 μM), apamin (3 μM) and INDO (10 μmol/l) Bradykinin-induced vasodilatation did not differ between groups or sex within group (Fig. 5a).

### Bradykinin-induced relaxation in placental chorionic plate vessels incubated with INDO and MgSO_4_

In the presence of INDO (10 μmol/l) and MgSO_4_ (0.2 mmol/l), bradykinin-induced vasodilatation was reduced in all groups, but did not differ between groups or sex within group (Fig. 5b).

### Bradykinin-induced relaxation in placental chorionic plate vessels incubated with TRAM-34 and apamin and MgSO_4_

In the presence of MgSO_4_ (0.2 mmol/l), TRAM-34 (1 μM) and apamin (3 μM) bradykinin-induced vasodilatation did not differ between group/sex within group (Fig. 5c).

### Vascular calcium concentration

**Fig. 6:**
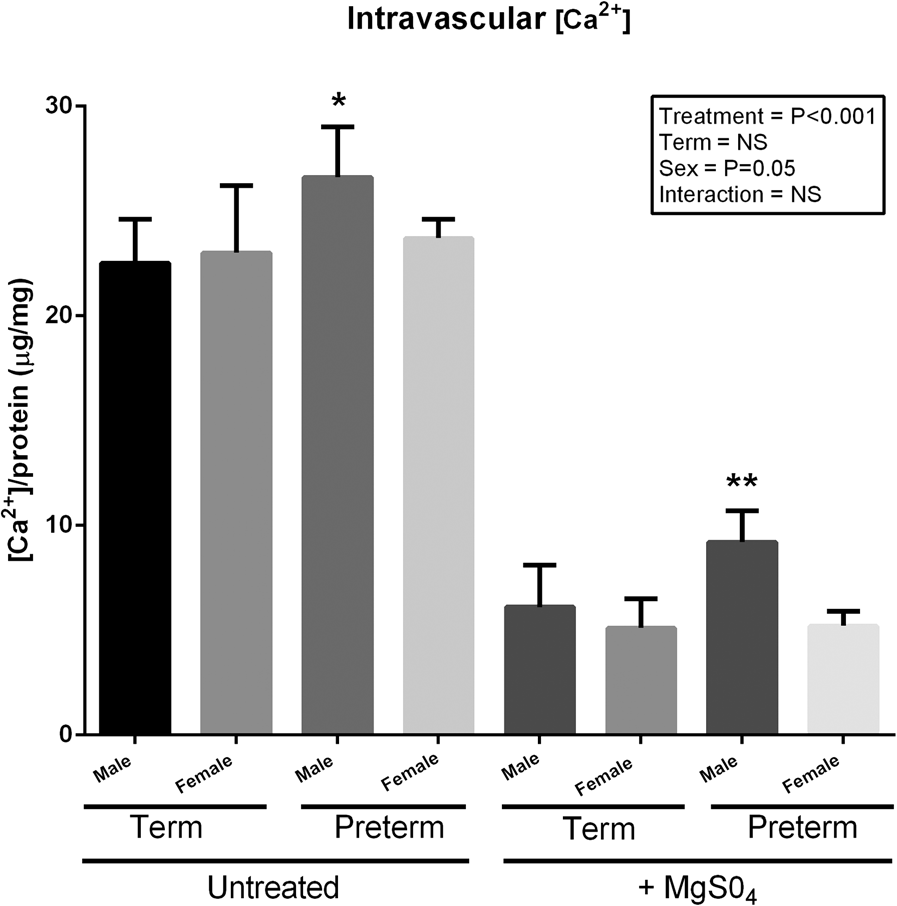
Biochemical analysis of basal intravascular [Ca^2+^]_i_ in vessels from male (*n* = 10) and female (*n* = 10) term pregnancies, male (*n* = 7) and female (*n* = 7) preterm pregnancies and male and female preterm pregnancies exposed to MgSO_4_ (0.2 mmol/l). Data was analysed using a repeated measures three-way ANOVA using treatment × term × sex as the main factors. *Denotes *P* < 0.05 between male and female preterm; **denotes *P* < 0.01 between male preterm and all other groups following post hoc analysis. All data are presented as means ± SEM

An overall effect of MgSO_4_ treatment was observed (main effect of treatment, *P* < 0.001, *F* = 9.87, *df* = 1) on vascular calcium concentration. MgSO_4_ significantly reduced vascular calcium concentration in male and female vessels of both groups (MgSO_4_ treated vs. MgSO_4_ untreated; main effect of term, *P* < 0.001, *F* = 5.35, *df* = 1). This equated to an average reduction in vascular calcium concentration of ~40 % (male preterm, 26.6 ± 2.4 vs. female preterm, 23.7 ± 0.9 μg/mg, male term, 22.5 ± 2.1 μg/mg and female term, 23 ± 2.2 μg/mg) following MgSO_4_ treatment. An overall effect of sex could also be observed (main effect of sex, *P* = 0.05, *F* = 18.15, *df* = 2) in vascular calcium concentrations. In male preterm placental vascular tissue, calcium concentrations were significantly higher when compared to female preterm vascular tissue following MgSO_4_ (male, 9.2 ± 1.5 μg/mg vs. female, 5.2 ± 0.7 μg/mg, male term, 6.1 ± 2.0 μg/mg, female term, 5.1 ± 1.4 μg/mg). No further effects of term or interactions were observed. Following post hoc analysis, intravascular calcium prior to MgSO_4_ treatment was significantly (*P* < 0.05) increased in male preterm vessels when compared to all other groups. Following MgSO_4_ treatment, male preterm vascular calcium concentrations were further significantly increased (*P* < 0.01) when compared to all other groups (Fig. 6).

## Discussion

This is the first report of the sex-specific and dose-dependent effect of MgSO_4_ on acute regulation of vascular reactivity in human placental chorionic plate vessels. Using small vessel pressure myography, we have demonstrated significant effects of sex on placental vasodilatation in response to MgSO_4_ treatment, specifically the vasculature of male preterm placentae having reduced vasodilatory responses to MgSO_4_ when compared with female placental vessels. Additionally, we provide evidence that these differences are likely, in part, due to competitive binding and inhibition of calcium channels and perturbation of EDHF pathways. Simultaneous analysis of vasomotor reactivity and [Ca^2+^]_i_ show that the specific mechanism of MgSO_4_-induced vasodilation is primarily caused by a decrease in vascular smooth muscle [Ca2+]_i_.

Consistent with published data, we observed a significant effect of MgSO_4_ on the modulation of vascular pathways related to smooth muscle function, specifically calcium homeostasis and EDHF function. This was manifest as a significant reduction in MgSO_4_-induced vasodilation in all vessels following inhibition of both NO and prostaglandin vasodilatory pathways. Although specific binding sites are not affected by MgSO_4_, competitive binding of non-specific calcium-binding sites are. Therefore, MgSO_4_ is likely to alter the efficacy of calcium to maintain a ‘normal’ maximum tension or vasodilation when applied to vascular smooth muscle by reducing the translocation of calcium into smooth muscle. Corroboratory evidence for this comes from our demonstration of a consistently higher vascular [Ca^2+^]_i_ in male preterm vessels when compared to female preterm placental chorionic plate vessels following incubation with MgSO_4_. This suggests that MgSO_4_ has a role in preventing free calcium uptake into the smooth muscle of these vessels, thus reducing basal vascular tone and promoting vasodilation. Moreover, it has been shown that Ca^2+^ blocks glutamate-activated Ca^2+^ and may stabilise fluctuations in blood pressure and heart rate and increase cerebral blood flow. Abad et al. previously reported MgSO_4_ treatment in preeclamptic pregnant women modified both Ca-ATPase activity and reduced lipid peroxidation concentrations in red blood cell membranes. They concluded that the inhibitory action of MgSO_4_ on lipid peroxidation accounts for an increase in Ca-ATPase activity to normotensive pregnancy concentrations. They hypothesised that this increases vasodilatation and potentially decreases maternal peripheral vascular resistance which may explain reduction in preeclamptic seizures [28]. The significant decrease in vasoconstriction and [Ca^2+^] in female vessels and decreased effects of MgSO_4_ observed on male placental vessels shows that a decreased capacity to regulate [Ca^2+^]_i_ homeostasis and impairment of Ca^2+^ transport are likely to play a role in the sex-specific differences of MgSO_4_-induced relaxation of placental vessels. This may be a key determinant in restoring optimal perfusion and nutrient supply to a foetus at risk of imminent preterm birth, whether consequent on preterm labour reducing placental perfusion or as a consequence of whichever pathological process initiated the need for ‘elective’ preterm delivery.

The current study has shown that vasodilatory responses to bradykinin in small placental chorionic plate vessels isolated from term and preterm were similar across group and sex when vascular inhibition of NO generation and PGI_2_ alone was performed. These results may indicate that in the absence of NO, PGI_2_ pathways may mediate vasodilatation. In the endothelium, MgSO_4_ has been shown to increase prostaglandin I_2_ production and increases NO production causing vasodilation  [29]. Although we report no difference in response to bradykinin in untreated vessels, analysis revealed differences in endothelium-dependent vasoresponsiveness to bradykinin in vessels from term vs. preterm groups following incubation with L-NAME, indomethacin, TRAM-34, apamin and MgSO_4_. The response to bradykinin in isolated vessels from term and preterm placentas shared some similarities, in that responses were unaffected by partial depolarisation. However, relaxation was significantly reduced by blockade of the NO and PGI_2_ pathways in male and female preterm placental vessels. This may indicate a reduced depolarization potential to EDHF in male preterm placental vessels.

Recommended clinical obstetric practice in the Capital and Coast District Health Board, Wellington, is to restrict the use of MgSO_4_ when delivery is anticipated at, or below, 30 weeks of gestation although use in other centres is more liberal [9, 30]. For this reason, we were able to study a population of women who delivered prematurely, yet did not receive MgSO_4_ as part of their routine care. Whether the sex-specific effects of MgSO_4_ would be greater at a lesser gestation is therefore unknown and may be difficult to answer given the widespread use of MgSO_4_ in this clinical population. Although, the mechanisms underlying the sex-specific vascular function in preterm placentae could not be fully elucidated in the current study. We have demonstrated that MgSO_4_ treatment at currently recommended therapeutic concentrations improves vascular responsiveness in placental vessels in both term and preterm placentae in a dose-dependent manner. Furthermore, this study provides direct evidence that the mechanism of MgSO_4_-induced relaxation in placental vessels involves a reduction in vascular [Ca2^+^]_i_ and more importantly is significantly attenuated in the placentae of preterm males compared to preterm females. We therefore promote the concept that it is no longer acceptable to pool placental data or make assumptions of placental function without acknowledging the specific sex of the placenta. Accordingly, future studies of placental function or perinatal intervention must be adequately powered to account for placental sex as an independent factor when considering experimental design and statistical analysis.

MgSO_4_ has been previously shown to increase vascular relaxation in the aorta, resistance vessels and placental perfusion in animal models and humans [31]. Similarly, our results suggest that perfusion of the preterm placenta is improved by maternal MgSO_4_ treatment and is likely due to increased plasma Mg resulting in competitive inhibition of calcium channels. This suggests that the effect of increased Mg^+^ competitively inhibiting Ca2^+^ entry into the smooth muscle is the primary causal factor in mediating vasodilation. Our findings support the hypothesis that the more specific action of MgSO_4_ on placental vascular function may primarily be governed by alterations in calcium homeostasis [32, 33]. Furthermore, the significant reductions in male preterm placental vasodilatory capacity is caused by EDHF-induced vasodilation and altered vascular [Ca2^+^]_i_ homeostasis and is likely to be the major mechanism involved in these sex-specific differences of MgSO_4_-induced relaxation in preterm placentae.

## Conclusions

In summary, we have examined the effects of MgSO_4_ on vascular tone in male and female placental vessels from term and preterm deliveries. Our results demonstrate that male and female foetuses may employ sex-specific survival strategies when presented with adverse maternal environments and associated pregnancy complications. Data presented here provide evidence that the placenta functions in a sex-specific manner and the overall neuroprotective effects of MgSO_4_ are most marked in preterm females as their placental bed is able to maximally relax in response to MgSO_4_, thus improving foetal nutrient delivery and gas exchange in the peri-partum period. This could be a major contributing factor in explaining that the sex-specific differences in perinatal morbidity and mortality may be due to sexually dimorphic placental adaptations during pregnancy and the relatively high neonatal morbidity and ongoing male disadvantage seen in Neonatal Units. However, clinical trials of MgSO_4_ have not yet addressed sex-specific long-term outcomes. In this era of pharmacogenetic where individualised drug therapy is the focus of much research interest, future advances in perinatal care need to be powered appropriately to incorporate the effect of foetal and therefore placental sex on outcomes of interest.

## Abbreviations

ATP: adenosine triphosphate
Ca^2+^: calcium ions
CP: cerebral palsy
EDHF: endothelial-derived hyperpolarizing factor
INDO: indomethacin
L-NAME: N_Ψ_-nitro-L-arginine methyl ester
MgSO_4_: magnesium sulphate
pEC80: concentration required to produce 80 %
PGH_2_: prostaglandin H2
PGI_2_: prostacyclin
PSS: physiological salt solution
TCB: tissue collection buffer
TRAM-34: Ca^2+^-activated K^+^ channel blocker
U46619: synthetic endoperoxide prostaglandin PGH_2_
